# Clinical Challenges in the Management of Complex Tooth Impaction and Transposition: A Case Series

**DOI:** 10.3390/children13070879

**Published:** 2026-06-30

**Authors:** Hana Omar AlBalbeesi, Eman Ibrahim Alshayea

**Affiliations:** 1 Department of Pediatric Dentistry and Orthodontics, Dental University Hospital, King Saud University, Riyadh 11545, Saudi Arabia; hbalbeesi@ksu.edu.sa; 2 Department of Pediatric Dentistry and Orthodontics, College of Dentistry, King Saud University, Riyadh 11545, Saudi Arabia

**Keywords:** cone-beam computed tomography; impacted teeth, orthodontic management, supernumerary teeth, transposed teeth

## Abstract

**Highlights:**

**What are the main findings?**

- Three paediatric patients with complex tooth impaction and transposition—including supernumerary-obstructed incisors, an impacted deciduous molar with premolar displacement, and a transposition complicated by root dilaceration—were successfully managed using individualised conservative orthodontic and surgical approaches, achieving favourable functional and aesthetic outcomes in all cases.
- Advanced three-dimensional imaging (CBCT), combined with staged surgical interventions, 2 × 4 appliances, bite-opening mechanics, and modified bracket biomechanics, was essential for accurate diagnosis, safe treatment planning, and minimising iatrogenic risks such as root resorption and adjacent tooth interference.

**What are the implications of the main findings?**

- Early interceptive orthodontic treatment guided by advanced imaging enables conservative management of complex eruption disturbances in growing patients, reducing the need for more invasive procedures and supporting normal dental development.
- Despite generally favourable outcomes, clinicians should anticipate potential complications—including incomplete root formation, residual spacing, and post-treatment restorative needs—underscoring the importance of long-term monitoring and multidisciplinary care in paediatric orthodontic cases.

**Abstract:**

Background/Objectives: Tooth impaction and transposition are developmental dental anomalies that pose significant challenges in orthodontics, oral surgery, and restorative dentistry. These anomalies disrupt normal occlusal development and complicate diagnosis and treatment planning because of altered anatomy, a higher risk of adjacent tooth resorption, associated pathology such as cyst formation, and biomechanical challenges during orthodontic intervention. Methods: Three paediatric patients with complex eruption disturbances, including impacted incisors, impacted primary molars, and transposed maxillary canines, were managed using individualised conservative orthodontic approaches. The cases involved obstruction by supernumerary teeth, ectopic eruption with space loss, and tooth transposition complicated by root dilaceration and malocclusion. Comprehensive clinical and radiographic assessment, including CBCT when indicated, guided treatment planning. Management strategies included staged surgical interventions, space maintenance using 2 × 4 appliances, bite opening, and controlled orthodontic traction with modified biomechanics to minimise the risk of root resorption and interference with adjacent teeth. Results: Favourable functional and aaesthetic outcomes were achieved in all cases, although certain limitations such as incomplete root development, residual spacing, localised restorative complications, and difficulty in correcting root angulation of dilacerated adjacent teeth were observed. Conclusions: Careful diagnosis using advanced imaging, combined with conservative orthodontic and surgical approaches, can achieve satisfactory functional and esthetic outcomes while minimising complications. Further longitudinal studies are recommended to evaluate long-term stability and optimise treatment protocols.

## 1. Introduction

Tooth impaction and transposition are developmental dental anomalies that pose significant challenges in orthodontics, oral surgery, and restorative dentistry. Tooth impaction is defined as the failure of a tooth to erupt into the dental arch within the expected timeframe, typically despite complete root formation and a fully developed root apex [1]. It should be noted that in clinical practice, the term ‘impaction’ is also applied to teeth whose eruption is physically obstructed prior to completion of root formation, as the mechanical barrier- rather than root maturity alone- constitutes the primary impediment to eruption [1]. This broader clinical application is relevant to the cases presented herein. Impacted teeth are relatively common, although prevalence varies considerably by tooth type and population. Third molars carry the highest impaction rates, with a pooled global prevalence of approximately 36.9% at the subject level [2]. Excluding third molars, maxillary canines and premolars also exhibit notable impaction rates, frequently necessitating intervention given their functional and aesthetic significance. Although impaction of the maxillary central incisor is uncommon, with a reported prevalence of less than 1%, it presents notable aesthetic, phonetic, and psychological consequences that require early detection and timely management [3,4,5].

In contrast, tooth transposition refers to the positional exchange of two adjacent teeth within the same quadrant, yielding an aberrant positional relationship with implications for occlusal function and dental aesthetics [6]. It carries a mean prevalence of 0.33%, most frequently involving the maxillary canine and first premolar [7]. Although often asymptomatic, both anomalies disrupt normal occlusal development and considerably complicate diagnosis and treatment planning, owing to altered dental anatomy, an elevated risk of adjacent tooth resorption, associated pathology such as cyst formation, and inherent biomechanical challenges during orthodontic intervention [6,8]. Moreover, impacted and transposed teeth can impair dental function, facial aesthetics, and psychosocial well-being, underscoring the imperative for effective and timely management. The aetiology of these anomalies is multifactorial, encompassing genetic predisposition, arch length deficiency, crowding, obstruction by supernumerary teeth or odontogenic lesions such as odontomas, trauma, and variations in eruption sequence or angulation [9,10]. Collectively, their complex aetiology, variable prevalence, and potential for serious sequelae reinforce the need for evidence-based, individualised treatment strategies. In this consecutive case series of paediatric patients, we present our clinical experience with the challenges encountered during management and highlight the value of conservative treatment approaches in addressing complex tooth impaction and transposition.

## 2. Materials and Methods

This manuscript presents a retrospective consecutive case series of three paediatric patients managed for complex tooth impaction and/or transposition at the Orthodontic Clinic, Dental University Hospital, King Saud University, between 2021 and 2025. All cases were treated by the same senior clinician (H.O.B.). Cases were selected on the basis of the following inclusion criteria: (i) paediatric patients presenting with complex eruption disturbances involving impaction, transposition, or a combination thereof; (ii) availability of complete clinical and radiographic records; and (iii) completion of active orthodontic treatment within the study period. Cases presenting without radiographic documentation or with incomplete records were excluded. The cases were selected to illustrate a spectrum of clinical complexity: Case 1 represents anterior impaction in the permanent dentition; Case 2 represents posterior impaction in the mixed dentition; and Case 3 represents transposition complicated by root dilaceration. CBCT was prescribed selectively, in accordance with the ALARA (As Low As Reasonably Achievable) principle [11], only when conventional radiography was insufficient to determine the three-dimensional relationship of impacted teeth to adjacent critical structures. This case series was reported in accordance with the CARE (CAse REport) guidelines. IRB approval was obtained and informed consent was provided by parents/guardians of all patients.

Three paediatric patients with complex eruption disturbances, including impacted incisors, impacted primary molars, and transposed maxillary canines, were managed using individualised conservative orthodontic approaches. The cases involved obstruction by supernumerary teeth, ectopic eruption with space loss, and tooth transposition complicated by root dilaceration and malocclusion. Comprehensive clinical and radiographic assessment guided treatment planning in all cases. Management strategies included staged surgical interventions, space maintenance using 2 × 4 appliances, bite opening, and controlled orthodontic traction with modified biomechanics to minimise the risk of root resorption and interference with adjacent teeth.

## 3. Case Presentations

### 3.1. Case 1

An 11-year-old boy with no significant medical or dental history presented to the orthodontic clinic with a chief complaint of an abnormal incisor shape since eruption, expressing concern regarding its appearance and seeking guidance on possible replacement options (Figure 1B). Clinical and radiographic examination, including periapical, orthopantomogram (OPG), cephalometric, and occlusal radiographs, revealed an impacted maxillary left central incisor (tooth #21) with two supernumerary teeth surrounding it and obstructing its normal eruption. The first supernumerary tooth had already erupted into the oral cavity, effectively replacing the central incisor, while the second supernumerary was located posterior to the impacted incisor #21 (Figure 1A–E and Figure 2A–D). The patient presented with a Class I molar relationship and a ¼-cusp canine relationship on the right, with a subdivision ½-cusp Class II relationship on the left, along with crowding in both the upper and lower arches and normal overjet and overbite. The erupted supernumerary was extracted to free the central incisor, which was subsequently surgically exposed and subjected to gradual forced eruption (Figure 3A,B). The second supernumerary was intentionally retained in situ until tooth #21 had been tractioned sufficiently to slide against it (Figure 4), thereby avoiding unnecessary trauma to the impacted tooth.

Once the second supernumerary became clinically visible behind the central incisor (Figure 5), it was extracted, and orthodontic alignment of tooth #21 was continued to close the diastema and remaining spaces (Figure 6A–E).

Following debonding (Figure 7), tooth #21 exhibited a short root that had failed to continue its development (Figure 8). A fixed retainer was placed in the lower arch and an Essex retainer in the upper arch. The patient was subsequently referred for gingivoplasty on the labial surface of tooth #21 to harmonise the gingival contours with the contralateral incisor. Active treatment duration was approximately 42 months. At the most recent follow-up, 15 months post-debonding, tooth #21 remained stable in the arch with no evidence of progressive root resorption.

### 3.2. Case 2

A 7-year-old boy with no significant medical or dental history was referred to the orthodontic clinic with an unerupted maxillary right second deciduous molar. Clinical examination (Figure 9A–E) and radiographic assessment—including periapical, OPG, cephalometric, and occlusal radiographs, as well as cone-beam computed tomography (CBCT) (Figure 10A–D)—confirmed impaction of the maxillary right second deciduous molar, mesial drifting of the maxillary right first permanent molar (tooth #16), and displacement of both maxillary right premolars (teeth #14 and #15) posterior to the impacted deciduous molar. 

The 2 × 4 appliance comprised 3M Unitek 0.022-inch pre-coated brackets bonded to maxillary incisors and maxillary first permanent molars, with a transpalatal arch for anchorage control. A 2 × 4 appliance was placed immediately, with a closed-coil spring extended from tooth #12 to tooth #16 to prevent further mesial drifting of tooth #16 and maintain arch length for the developing premolars. The impacted right maxillary second deciduous molar was surgically removed, and the patient was kept under close observation until tooth #14 erupted (Figure 11). Alignment of tooth #14 and de-rotation of tooth #15 were subsequently performed (Figure 12A,B). Following alignment, a post-treatment complication arose in which tooth #15 sustained a crown fracture and developed a carious lesion (Figure 13). The crown fracture of tooth #15 was attributed to trauma from mastication of hard food, as reported by the patient’s guardian. Caries subsequently developed at the fracture margin, likely facilitated by compromised enamel integrity and difficulty maintaining adequate oral hygiene at the fracture site during orthodontic treatment. Structural vulnerability secondary to ectopic eruption may have predisposed tooth #15 to this complication. The tooth was restored with composite resin, and a definitive crown restoration is planned upon completion of skeletal growth at approximately 18 years of age. The case was debonded, and a fixed retainer in the lower arch along with an Essex retainer in the upper arch were placed (Figure 14 and Figure 15A–E). Active treatment duration was approximately 48 months. At the most recent follow-up, 15 months post-debonding, teeth #14 and #15 were well-aligned with stable contacts, and the restored tooth #15 was being monitored for caries recurrence.

### 3.3. Case 3

A 10-year-old boy with no significant medical or dental history was referred to the orthodontic clinic, presenting with unerupted maxillary permanent canines. Clinical examination revealed a Class II Division 2 malocclusion associated with a traumatic deep bite (100% overbite), a reduced overjet of 1 mm, and lower arch crowding (Figure 16A–E). Radiographic assessment demonstrated transposition of tooth #23, with its root apex positioned superior to teeth #24 and #25 and the crown tip of #23 overlapping tooth #24. The presentation was further complicated by root dilaceration of tooth #24 (Figure 17A,B).

Comprehensive radiographic investigations, including OPG, cephalometric, occlusal, periapical radiographs, and CBCT, were performed to evaluate the spatial relationship and proximity of the transposed tooth to adjacent structures, ensuring that planned orthodontic movements would not compromise surrounding teeth or induce root resorption. An anterior bite plane was initially placed to relieve the traumatic deep bite. Concurrently, fixed appliances were bonded in the lower arch to alleviate crowding and facilitate the eruption of tooth #23. A 2 × 4 fixed appliance was subsequently placed in the maxillary arch, and controlled bodily movement of tooth #23 was initiated in a mesial direction to guide it toward its correct position. To prevent root interference between tooth #23 and the dilacerated root of tooth #24, the bracket on tooth #24 was intentionally inverted to direct its root away from the path of tooth #23 during alignment (Figure 18A–F). Figure 19 demonstrated the dilaceration of the root of tooth #24 in two periapical radiographs, during the course of treatment and at completion, both of which confirm the angular deviation of the root at the dilaceration point and explain the limitation of further root angulation correction without endodontic surgical intervention. Treatment was completed with Class I molar and canine relationships. Residual spaces distal to the lateral incisors (#12 and #22) were maintained with the intention of closure via composite build-ups through referral to restorative dentistry; however, the patient’s guardian declined the proposed restorative intervention, opting to maintain the spaces without closure. Tooth #24 was slightly rotated to aid in space camouflage and improve overall aesthetics. Further mesialisation of tooth #24 was constrained by its root dilaceration, with apical modification via endodontic surgical intervention considered as an option if required in the future. Debonding was performed following placement of a fixed retainer in the lower arch and an Essex retainer in the upper arch (Figure 20A–F). Active treatment duration was approximately 48 months. The final orthodontic outcome, Class I molar and canine relationships, was stable at the most recent review, 13 months post-debonding, with residual spacing maintained as planned.

## 4. Discussion

Precise diagnosis is a fundamental prerequisite for the safe and effective management of complex eruption anomalies. Three-dimensional imaging, such as CBCT, affords superior three-dimensional localization of impacted or transposed teeth compared with conventional radiographs. Such enhanced diagnostic capability improves prognostic accuracy and supports effective biomechanical planning, particularly for teeth occupying challenging positions or lying in close proximity to critical anatomical structures [11]. The management of impacted and transposed teeth in paediatric patients remains a complex and technique-sensitive domain of contemporary orthodontic care [12]. The present case series highlights the considerable variability in clinical presentation, aetiology, and treatment challenges associated with these anomalies, while reinforcing the value of early diagnosis and conservative, individualised management approaches.

Impaction of anterior teeth—particularly maxillary central incisors—although relatively uncommon, carries significant aesthetic, psychological, and functional implications. In Case 1, impaction of tooth #21 was primarily attributable to the presence of supernumerary teeth, a well-documented aetiological factor in this context [5]. The sequential removal of supernumerary teeth, combined with controlled orthodontic traction, facilitated successful eruption and alignment of the impacted incisor. This staged approach effectively minimised surgical trauma and preserved surrounding structures, consistent with previously recommended interceptive protocols [13]. It is acknowledged that in selected cases, particularly younger patients with greater eruptive potential and less urgent aesthetic demands, a more conservative approach involving removal of the obstructing supernumerary teeth followed by a period of observation for spontaneous eruption may be appropriate [13]. In the present case, however, the patient’s aesthetic concerns and the extended duration of non-eruption made active orthodontic traction the preferred option.

The second supernumerary tooth was intentionally retained in situ during the initial phase of traction to provide a solid osseous scaffold posterior to the impacted tooth, thereby facilitating its controlled eruption and avoiding excessive movement through a large surgical void. Although retaining a supernumerary in proximity to an erupting tooth theoretically carries a risk of iatrogenic root resorption through mechanical obstruction, several lines of evidence argue against this mechanism in the present case. Surgical exploration confirmed that the second supernumerary was oriented parallel to the long axis of tooth #21, with no contact or overlap at the root apex level, a relationship subsequently confirmed clinically when the supernumerary emerged posteriorly rather than coronally to the incisor. Furthermore, comparative radiographic assessment revealed only minimal and symmetrical apex shortening, inconsistent with the pattern of root resorption expected from direct mechanical pressure. Taken together, these findings support the conclusion that the short root of tooth #21 reflects a pre-existing compromise in root formation secondary to prolonged obstruction and eruption delay, rather than an iatrogenic consequence of the treatment approach adopted. This interpretation is consistent with evidence indicating that impacted incisors may exhibit compromised root formation, particularly when eruption is delayed or obstructed over an extended period [14]. The short root observed at debonding therefore underscores the importance of early diagnosis and timely intervention in similar cases, as well as the need for long-term clinical monitoring and judicious force application to mitigate the cumulative risks of root resorption, ankylosis, and iatrogenic damage, as highlighted in recent literature on the biomechanical and surgical management of impacted teeth [15].

Case 2 illustrates a comparatively uncommon clinical scenario involving an impacted maxillary second deciduous molar, resulting in mesial drift of the permanent first molar and displacement of the developing premolars. Although the delayed eruption of tooth #55 in a 7-year-old child may appear consistent with simple retention in isolation, CBCT examination (Figure 10D) confirmed a genuine impaction: the sequential vertical stacking of tooth #55, tooth #14, and tooth #15 within the alveolus, combined with the obstruction created by the mesially drifted tooth #16, constituted a physical barrier precluding normal eruption. This presentation is therefore classified as true impaction rather than developmental delay. Early intervention with a 2 × 4 appliance proved instrumental in preserving arch length and preventing further space loss, in keeping with established principles of interceptive orthodontics [16]. Surgical removal of the impacted primary molar facilitated spontaneous eruption and subsequent orthodontic alignment of the premolars, as supported by previous clinical reports [17].

An alternative treatment strategy that merits consideration is the extraction of tooth #16, particularly when structurally compromised, combined with facilitated mesialisation of the maxillary second molar. This approach, supported by evidence demonstrating over 90% predictive success in the maxilla when the permanent second molar is at Demirjian developmental stages D or E [18], may reduce overall treatment complexity and avoid the biomechanical challenges associated with premolar alignment. In the present case, this option was discussed with the family but declined, and the approach adopted was therefore guided by parental preference in addition to clinical considerations.

The occurrence of enamel fracture and caries in tooth #15 following alignment represents a notable post-treatment complication. The fracture was attributed to trauma from mastication of hard food, as reported by the patient’s guardian, with caries subsequently developing at the fracture margin secondary to compromised enamel integrity and impaired oral hygiene maintenance at the fracture site during active orthodontic treatment. Teeth that have undergone ectopic eruption or prolonged displacement may additionally harbour areas of enamel hypomineralization or inherent structural weakness that predispose them to fracture under normal masticatory loads [5,19], which may have further contributed to this complication. This case therefore highlights the importance of proactive preventive counselling—encompassing dietary modification and reinforced oral hygiene instruction—as well as regular clinical monitoring throughout treatment, to enable early detection of structural changes and timely restorative intervention in paediatric orthodontic patients.

Tooth transposition, as illustrated in Case 3, exemplifies one of the most clinically demanding orthodontic anomalies, owing to the associated risks of root interference, resorption, and biomechanical constraints. The transposition of tooth #23, compounded by root dilaceration of tooth #24 and a severe traumatic deep bite, necessitated meticulous interdisciplinary planning and precise clinical execution. Advanced imaging, particularly CBCT, has been shown to significantly enhance diagnostic accuracy and treatment safety by enabling precise three-dimensional assessment of tooth position and proximity to adjacent structures [20]. The decision to correct the transposition through controlled bodily movement, rather than accepting the transposed position or resorting to extraction, reflects a conservative philosophy supported in the literature when anatomically and biomechanically feasible [21]. The decision to actively correct the transposition rather than accept it and treat the teeth in their transposed positions was informed by the patient’s age (providing adequate biological potential for movement), the partial nature of the transposition (which had not progressed to complete positional exchange), and the expressed preference of the patient’s guardian following informed discussion of both options. The literature supports either approach depending on clinical context [21,22], and the choice in this case reflects an individualised decision-making process consistent with evidence-based principles. The individualised biomechanical modification employed in this case specifically, bracket inversion to prevent root collision, demonstrates the critical importance of tailored mechanics in minimising iatrogenic risk [22]. It is important to acknowledge that the final panoramic radiograph demonstrates residual proximity between the roots of tooth #23 and tooth #24. This is a recognised consequence of the anatomical constraint imposed by the root dilaceration of tooth #24, which limited the extent to which root angulation could be corrected through orthodontic mechanics alone. The patient has been counselled regarding this limitation, and long-term radiographic monitoring is planned. Should further root angulation correction be deemed necessary, endodontic surgical intervention -specifically apical modification- would need to be considered prior to further orthodontic movement. Nonetheless, the persistence of residual spacing secondary to anatomical constraints underscores the necessity of balancing ideal treatment objectives with biological limitations and patient preferences.

CBCT was prescribed selectively, in accordance with the ALARA (As Low As Reasonably Achievable) principle, only when conventional two-dimensional radiography was insufficient to determine the precise three-dimensional position of impacted or transposed teeth relative to adjacent anatomical structures [11,20,23]. In Case 1, the SLOB parallax technique applied to periapical and occlusal radiographs provided adequate localisation of the supernumerary teeth and the impacted central incisor. CBCT was therefore not indicated at the initial stage. In Cases 2 and 3, the complexity of the spatial relationships and the proximity to critical structures necessitated CBCT imaging, which was performed accordingly.

Across all three cases, early diagnosis and timely interceptive treatment emerged as pivotal determinants of favourable outcomes. The use of simplified fixed appliances, such as the 2 × 4 system, in conjunction with adjunctive surgical procedures has been widely advocated as an effective strategy for managing eruption disturbances in growing patients [16,24]. These approaches support normal dental development while reducing the likelihood of more invasive interventions at a later stage. A primary limitation of the present case series is the relatively short post-treatment follow-up period. Long-term stability data, particularly with regard to root length maintenance in Case 1 and the residual root proximity in Case 3, are not yet available. Extended follow-up evaluations of these cases are planned and will be reported in a subsequent publication. Clinicians managing similar presentations should be cognisant of the need for long-term radiographic monitoring. Despite the generally favourable outcomes achieved, several limitations were noted, including incomplete root development, residual spacing, and treatment-related complications such as tooth fracture. Furthermore, the degree of patient and parental acceptance of adjunctive procedures may substantially influence the final treatment outcome. These observations align with previous reports underscoring the inherently unpredictable nature of managing impacted and transposed teeth [6,8].

## 5. Conclusions

This case series illustrates the clinical complexity and variability inherent in the management of tooth impaction and transposition in paediatric patients. The cases presented suggest that individualised, multidisciplinary approaches, guided by advanced imaging and conservative orthodontic principles, may achieve satisfactory functional, aesthetic, and psychosocial outcomes in selected presentations. However, given the limited sample size and the absence of long-term follow-up data, these findings should be interpreted with caution and cannot be generalised beyond the cases described. The complications observed in this series, including incomplete root development, residual spacing, and post-alignment tooth fracture, underscore the unpredictable nature of managing these anomalies and the importance of realistic pre-treatment counselling. Further prospective studies with adequate follow-up periods are warranted to establish robust evidence-based protocols for the management of complex eruption disturbances in growing patients.

## Figures and Tables

**Figure 1:**
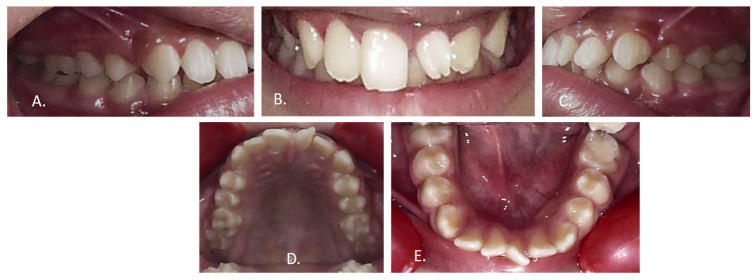
Pre-treatment intra-oral photographs. (**A**) Right lateral view. (**B**) Frontal view. (**C**) Left lateral view. (**D**) Upper occlusal view. (**E**) Lower occlusal view.

**Figure 2:**
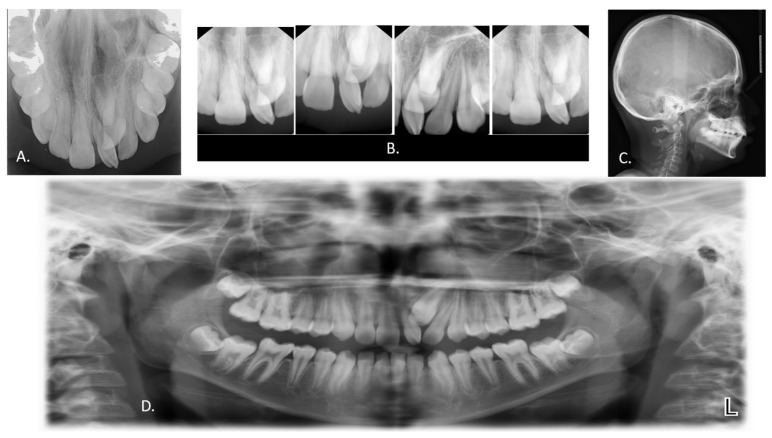
Pre-treatment radiograph. (**A**) Occlusal. (**B**) Periapicals. (**C**) Lateral Cephalogram. (**D**) Panoramic.

**Figure 3:**
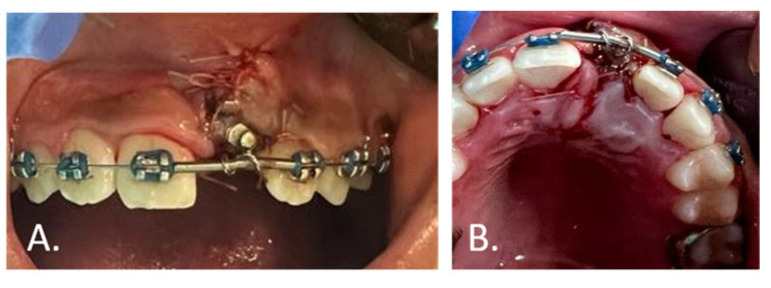
Surgical exposure and forced eruption of tooth # 21. (**A**) Frontal view. (**B**) Upper occlusal view.

**Figure 4:**
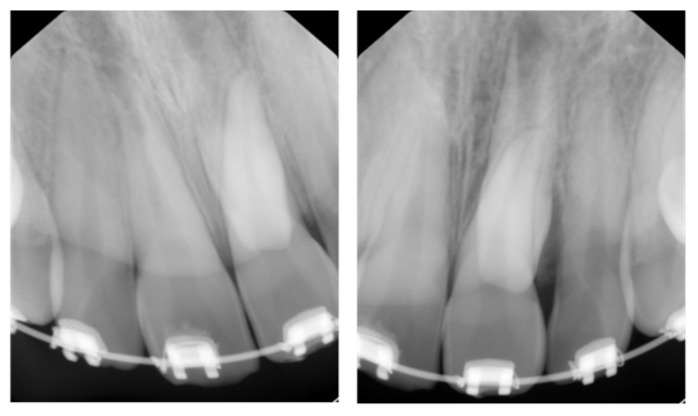
Periapical radiograph demonstrating the forced eruption of tooth #21 and the clinical emergence of the second supernumerary tooth posterior to the central incisor. Buccolingual localisation of the supernumerary was determined using the SLOB parallax technique applied to two periapical radiographs taken at different horizontal angulations, confirming its palatal position relative to tooth #21.

**Figure 5:**
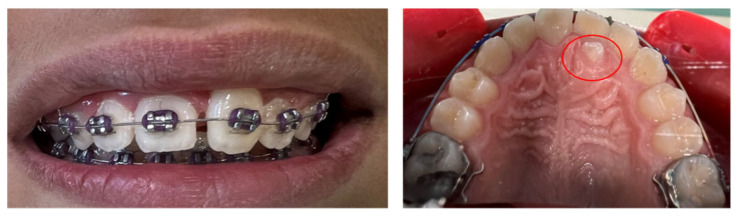
Intra-oral photographs demonstrating the forced eruption of tooth 21 and the emergence of a second supernumerary tooth behind the central incisor.

**Figure 6:**
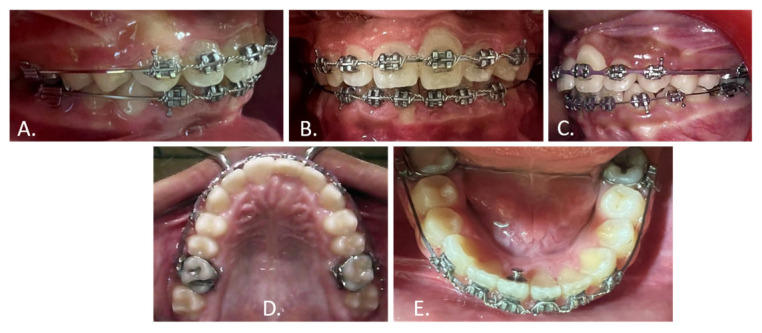
Intraoral photographs taken during the alignment process, following the removal of the second supernumerary tooth. (**A**) Right lateral view. (**B**) Frontal view. (**C**) Left lateral view. (**D**) Upper occlusal view. (**E**) Lower occlusal view.

**Figure 7:**
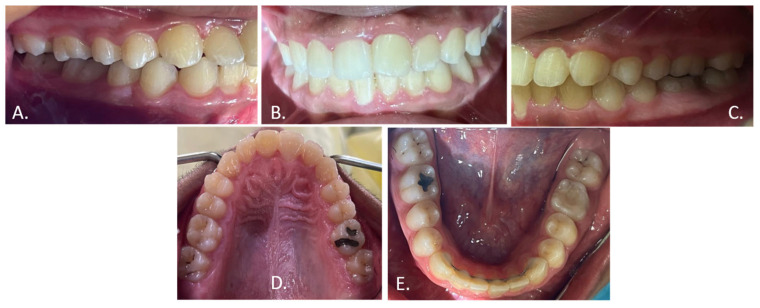
Postoperative intraoral photographs demonstrating successful alignment of tooth #21 with intact incisal margins and harmonious arch form. (**A**) Right lateral view. (**B**) Frontal view. (**C**) Left lateral view. (**D**) Upper occlusal view. (**E**) Lower occlusal view.

**Figure 8:**
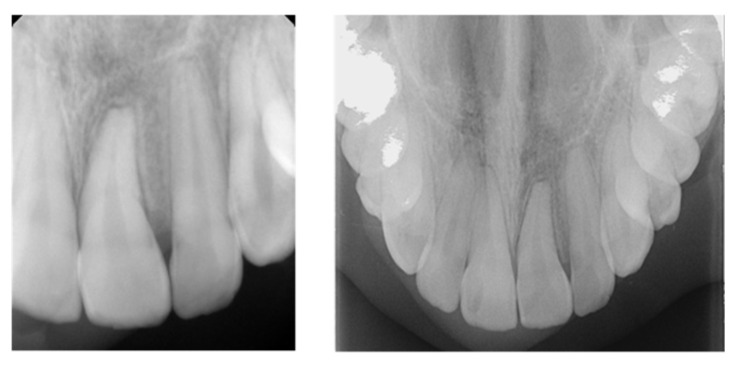
Periapical and occlusal radiographs demonstrating tooth #21 with a short root.

**Figure 9:**
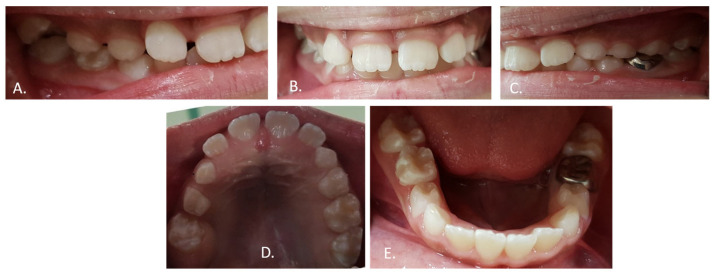
Pre-treatment intra-oral photographs. (**A**) Right lateral view. (**B**) Frontal view. (**C**) Left lateral view. (**D**) Upper occlusal view. (**E**) Lower occlusal view.

**Figure 10:**
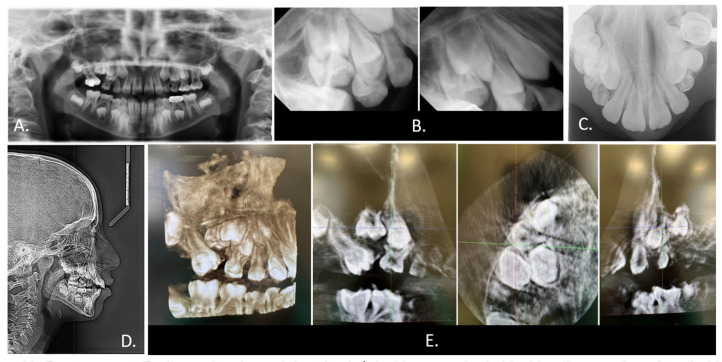
Pre-treatment radiographs showed that the 2nd deciduous molar is blocking the eruption of teeth 14 and 15. (**A**) Panoramic. (**B**) Periapicals. (**C**) Occlusal. (**D**) Lateral Cephalogram. (**E**) CBCT.

**Figure 11:**
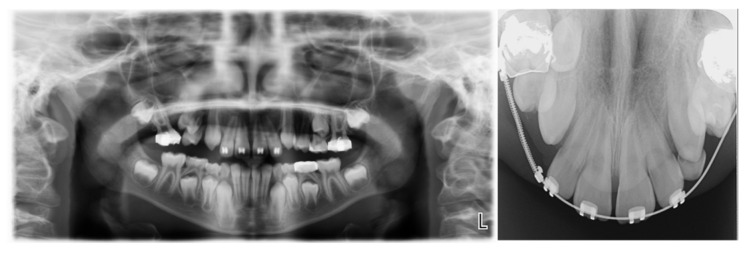
Radiographic findings during treatment with a 2 × 4 appliance to prevent mesial drifting of tooth #16, align the incisors, and maintain space for teeth #15 and #14 after the removal of the second deciduous molar.

**Figure 12:**
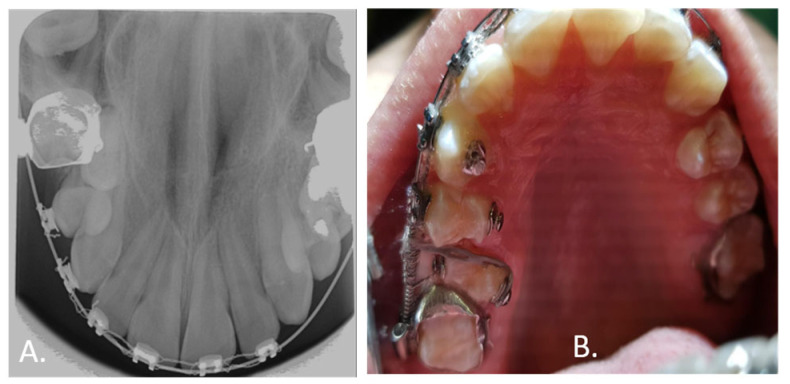
Progress findings during alignment of #14 and de-rotation of #15. (**A**) Occlusal radiographic view. (**B**) Occlusal clinical view.

**Figure 13:**
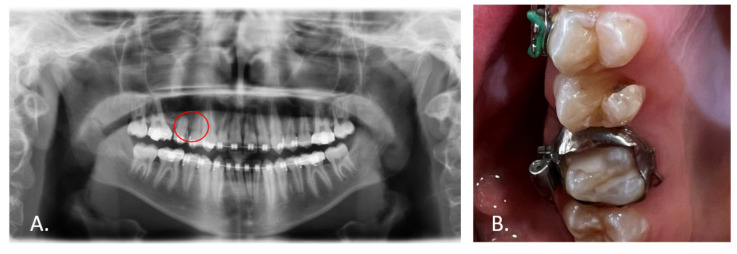
Post-alignment complication of tooth #15 showing crown fracture and development of a carious lesion. (**A**) Panoramic radiograph. (**B**) Occlusal clinical view.

**Figure 14:**
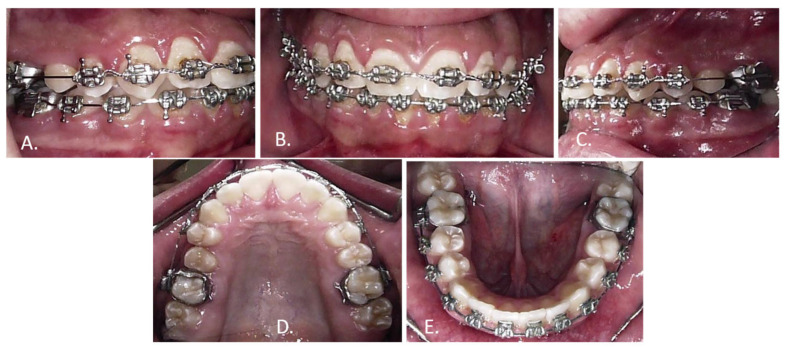
Progress intra-oral photographs before debonding. (**A**) Right lateral view. (**B**) Frontal view. (**C**) Left lateral view. (**D**) Upper occlusal view. (**E**) Lower occlusal view.

**Figure 15:**
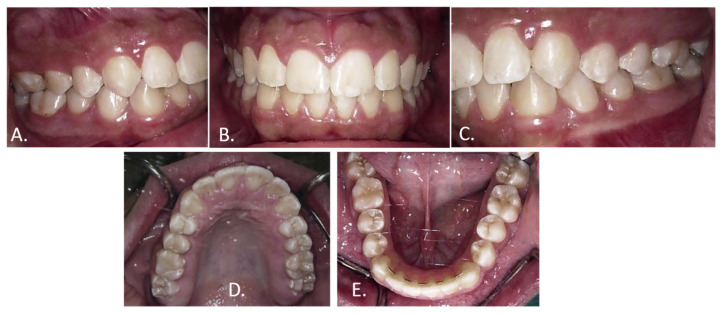
Post-treatment intra-oral photographs. (**A**) Right lateral view. (**B**) Frontal view. (**C**) Left lateral view. (**D**) Upper occlusal view. (**E**) Lower occlusal view.

**Figure 16:**
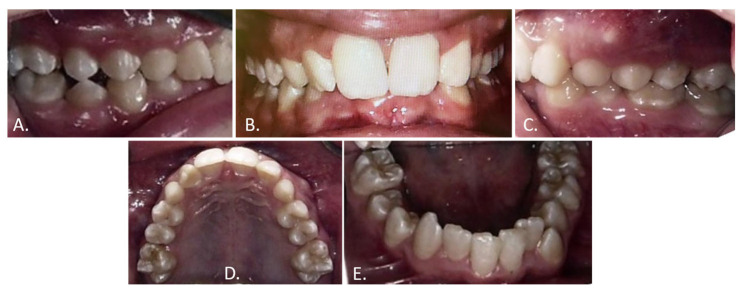
Pre-treatment intra-oral photographs. (**A**) Right lateral view. (**B**) Frontal view. (**C**) Left lateral view. (**D**) Upper occlusal view. (**E**) Lower occlusal view.

**Figure 17:**
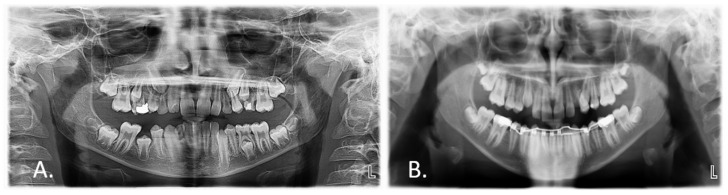
Pre-treatment panoramic radiographs showed transposition of tooth #23 and root dilaceration of tooth #24. (**A**). Before exfoliation of deciduous maxillary canines. (**B**) After exfoliation of deciduous maxillary canines.

**Figure 18:**
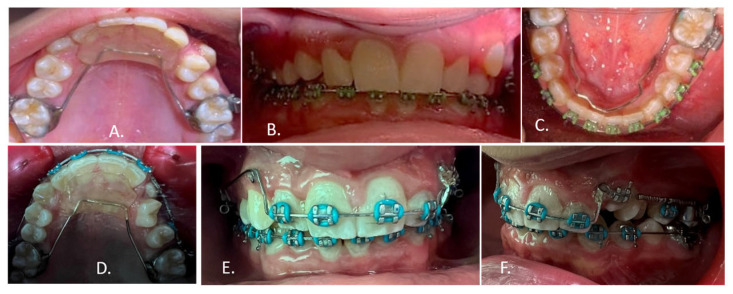
Progress intra-oral photographs. (**A**) Placement of an anterior bite plane to relieve the traumatic deep bite. (**B**,**C**) Fixed appliance was bonded in the lower arch to alleviate crowding. (**D**–**F**) Upper occlusal, frontal and left side views during the placement of a 2 × 4 fixed appliance in the maxillary arch.

**Figure 19:**
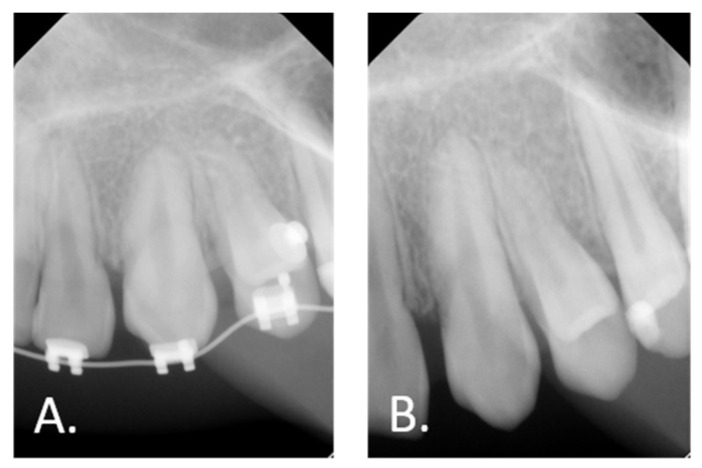
Periapical radiographs demonstrate the dilaceration of the root of tooth #24. (**A**) During the course of treatment. (**B**) At completion of treatment.

**Figure 20:**
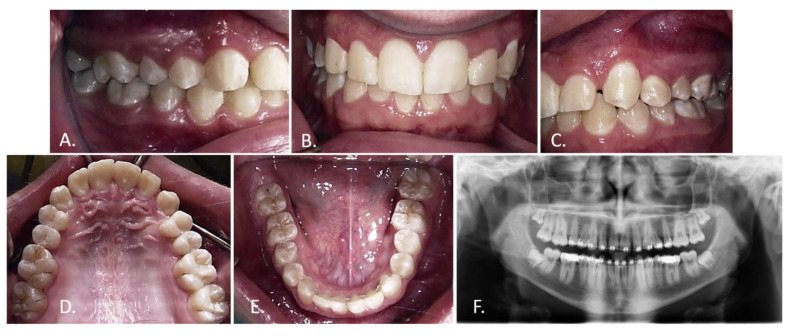
Post-treatment intra-oral photographs and radiograph. (**A**) Right lateral view. (**B**) Frontal view. (**C**) Left lateral view. (**D**) Upper occlusal view. (**E**) Lower occlusal view. (**F**) Panoramic radiograph.

## Data Availability

The data presented in this study are available upon request from the corresponding author due to ethical reasons.

## Abbreviations

The following abbreviations are used in this manuscript:

OPG	OrthoPantomoGram
CBCT	Cone-Beam Computed Tomography
